# Molecular Dynamics Simulations and Dynamic Network Analysis Reveal the Allosteric Unbinding of Monobody to H-Ras Triggered by R135K Mutation

**DOI:** 10.3390/ijms18112249

**Published:** 2017-10-26

**Authors:** Duan Ni, Kun Song, Jian Zhang, Shaoyong Lu

**Affiliations:** Key Laboratory of Cell Differentiation and Apoptosis of Chinese Ministry of Education, Department of Pathophysiology, School of Medicine, Shanghai Jiao Tong University, Shanghai 200025, China; niduan11@sjtu.edu.cn (D.N.); sk1354910551@163.com (K.S.); methane02@163.com (J.Z.)

**Keywords:** H-Ras, molecular dynamics (MD) simulations, allosteric pathway, allosteric network, allostery, R135K mutation

## Abstract

Ras proteins, as small GTPases, mediate cell proliferation, survival and differentiation. Ras mutations have been associated with a broad spectrum of human cancers and thus targeting Ras represents a potential way forward for cancer therapy. A recently reported monobody NS1 allosterically disrupts the Ras-mediated signaling pathway, but its efficacy is reduced by R135K mutation in H-Ras. However, the detailed mechanism is unresolved. Here, using molecular dynamics (MD) simulations and dynamic network analysis, we explored the molecular mechanism for the unbinding of NS1 to H-Ras and shed light on the underlying allosteric network in H-Ras. MD simulations revealed that the overall structures of the two complexes did not change significantly, but the H-Ras–NS1 interface underwent significant conformational alteration in the mutant Binding free energy analysis showed that NS1 binding was unfavored after R135K mutation, which resulted in the unfavorable binding of NS1. Furthermore, the critical residues on H-Ras responsible for the loss of binding of NS1 were identified. Importantly, the allosteric networks for these important residues were revealed, which yielded a novel insight into the allosteric regulatory mechanism of H-Ras.

## 1. Introduction

Ras proteins, a series of GTPases [1], play key roles in the regulation of cell proliferation, cell survival as well as cell motility [2,3], and thus are closely related to tumorigenesis [4,5,6,7,8,9]. Ras-activating mutations can be found in about 30% of cancers in human beings, especially those highly malignant and resistant to traditional therapies [10,11,12,13,14,15]. Onco-mutations of Ras are observed in cancer [11,15,16,17,18,19], particularly in pancreatic adenocarcinomas, and the occurrence of K-Ras mutations in patients reaches as high as 90% [20,21,22,23,24]. Therefore, targeting Ras proteins has already become one of the latest hot spots in drug design for anti-cancer purposes [11,25,26,27].

Ras can be characterized as the activator in the RAS-RAF-MEK-ERK pathway [28,29,30,31], and the activation of the downstream Raf proteins requires the dimerization of Ras [30,31]. In their active forms, Ras-GTP complexes can form dimers with each other [24,32,33], which can subsequently recruit the Raf kinase. Due to the close distance between the Ras dimer, the recruited Raf are in close proximity and dimerize [24], and the Raf dimer can further activate the downstream MEK-ERK pathway. The binary switch function of Ras mainly depends on the way they exist. Ras can be divided into GDP-bound inactive forms, and GTP-bound active forms [34]. Considerable effort is currently invested to the development of inhibitors directly targeting the GDP/GTP binding site [35]. Until now, however, little progress has been reported in the search for therapeutic agents targeting the primary site because of the high affinity of the binding of GDP or GTP [36], which reduces the potential molecules to failure in the competition with GDP or GTP. With the previous failure, and together with the advances of structural biology and allostery, interacting with the allosteric sites of Ras has been established as an alternative method for the Ras-targeting treatments [11,28].

Allosteric Ras therapies mainly focus on the binding of therapeutic agents to the allosteric sites [26,28,36]. By targeting the allosteric sites, the dimerization of Ras proteins or the proteins interaction in the signaling pathway is disrupted, and consequently, the signal transduction is regulated [37,38,39]. Recently, Spencer-Smith et al. synthesized a binding protein NS1 (monobody) targeting the allosteric site on Ras. Binding of NS1 inhibits Ras dimerization as well as the downstream CRAF-BRAF heterodimerization, therefore inhibiting the cell growth-signaling pathway [40]. However, in vitro experiments showed that R135K mutation in H-Ras greatly reduced the affinity of the binding of the NS1 to H-Ras, which significantly weakened its efficacy [40]. Until now, the underlying mechanism for the affinity change is still unclear. Molecular dynamic (MD) simulations can reveal the landscapes of the changes in proteins after mutations [41,42,43,44,45,46], and they have been frequently applied to study the on-protein conformational changes in response to mutations or modifications [47,48], especially for the ones related to cancer or tumorigenesis [49,50,51,52,53,54]. Hence, we employed the MD simulations and in silico analysis to explore the H-Ras and NS1 complex system. By carrying out 200 ns MD simulations, and analyzing a series of statistical values, we demonstrated that at the critical point mutation R135K disrupted the binding of NS1 to H-Ras, and there was an underlying allosteric mechanism responsible for such effect. Moreover, by analyzing the residue topology, we further identified a potential allosteric network in this complex system, which will offer a guidance for future drug development related to Ras proteins.

## 2. Results and Discussion

### 2.1. RMSD and RMSF Analysis

**Figure 1:**
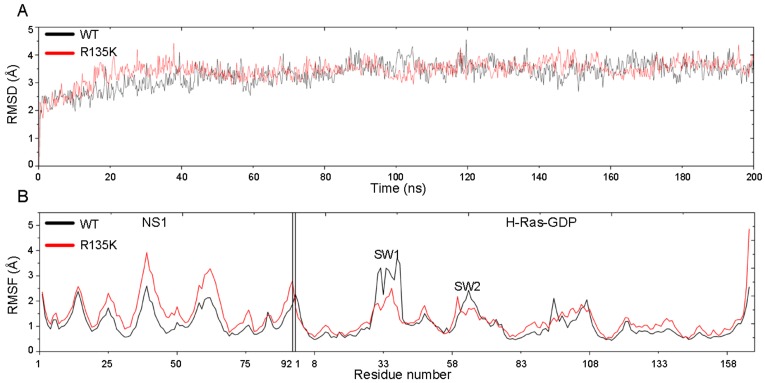
(**A**) RMSD of the wild type and R135K H-Ras–NS1–GDP complexes in 200 ns simulation; (**B**) The Cα atoms RMSF of the wild type and mutation system in 200 ns simulation (Residue 1–92 was for NS1, and 1–166 was for H-Ras–GDP).

200 ns MD simulations were carried out for both the wild type and R135K H-Ras–NS1 complexes. To quantify the dynamic conformational changes throughout the simulations, the Cα atoms root-mean-square deviation (RMSD) of simulated snapshots relative to the original crystal structure were calculated. As shown in Figure 1A, the two systems became relatively stable after 50 ns simulations, which implied that the dynamic process reached equilibrium states, and thus the following analysis was focused on the period of 50–200 ns simulations. During the last 150 ns, the RMSD values for the wild type and mutation complexes were 3.48 ± 0.31 Å and 3.53 ± 0.25 Å, respectively, which showed no significant difference. This suggested that the overall conformation of H-Ras^R135K^–NS1 complex adopted a similar topology to the wild type complex.

Root-mean-square fluctuation (RMSF) was calculated to reveal the differences in the fluctuation of local regions for the two systems. Generally, the mutant displayed a higher RMSF, especially for the NS1 part, which showed that the mutated system was less stable, particularly for NS1. Also, loop SW1 in H-Ras was relatively flexible and had high RMSF in both systems, whose conformation changed greatly after R135K mutation. Since during simulations, NS1 binding was unfavored in the R135K system, it would gradually disassociate from H-Ras, which would be discussed later. Unbinding of NS1 would relieve the constraint stemmed from its interaction with H-Ras. Considering its peptide nature, which mainly consisted of beta sheets, and the reduced restriction, NS1 unsurprisingly displayed higher flexibility and RMSF after mutation.

### 2.2. DCCM and PCA Analysis

**Figure 2:**
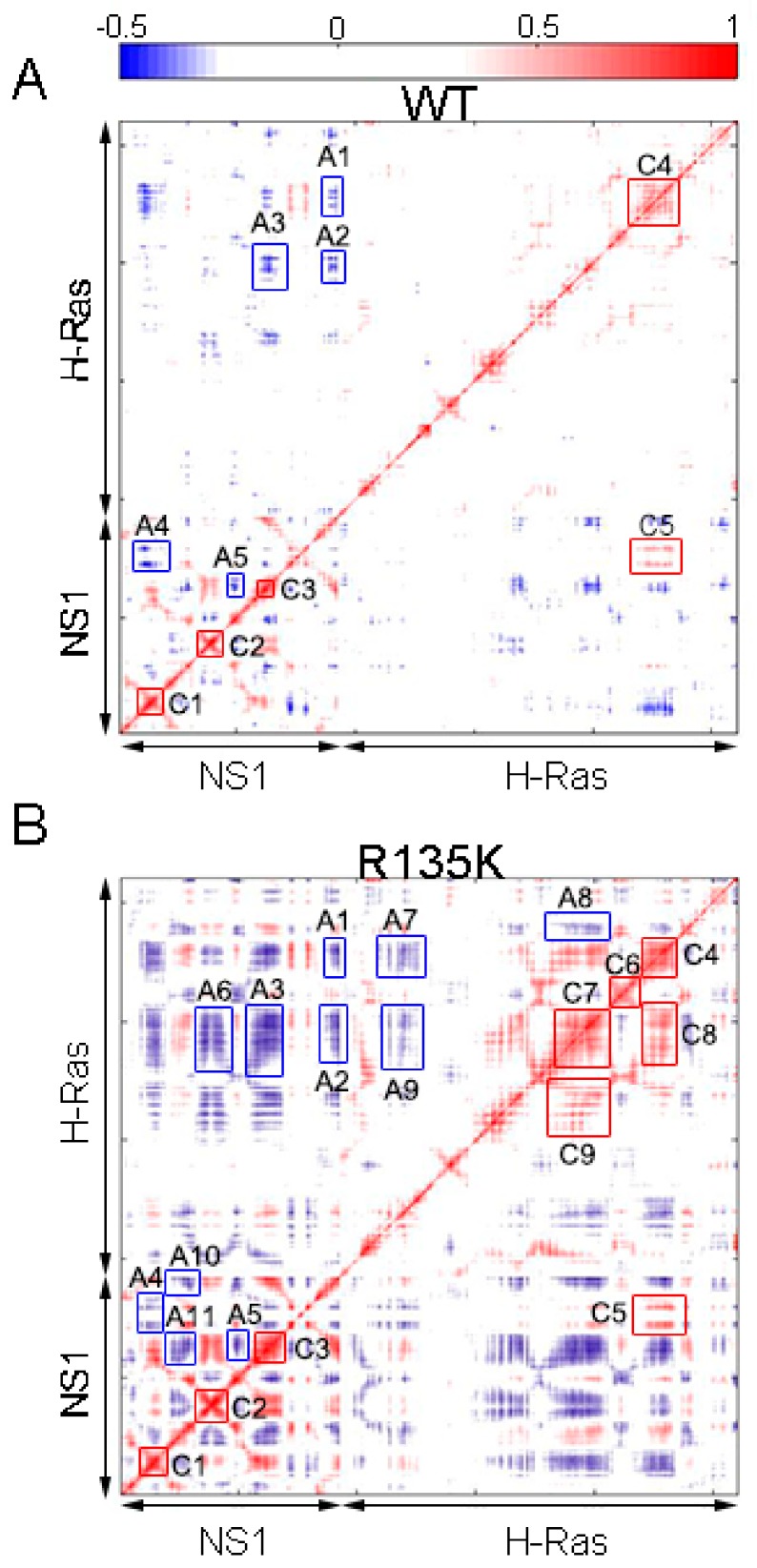
Dynamic cross-correlation matrices of wild type complex (**A**) and R135K (**B**) H-Ras–NS1 complex. Red stands for correlation and blue stands for anticorrelation. Correlated motions whose absolute values were smaller than 0.3 were neglected.

To analyze the effect of the mutation on the intrachain motions in the complex system, Dynamic cross-correlation matrix (DCCM) were calculated for each residue within two complexes, and it showed that R135K mutation resulted in an increase of correlated motions, especially for residues near the interface between NS1 and H-Ras (Figure 2). The higher correlation indicated that there were more fluctuation and residues interaction within the complex due to the mutation. Moreover, several newly formed correlations within NS1 or H-Ras reflected that the original allosteric network was disturbed.

**Figure 3:**
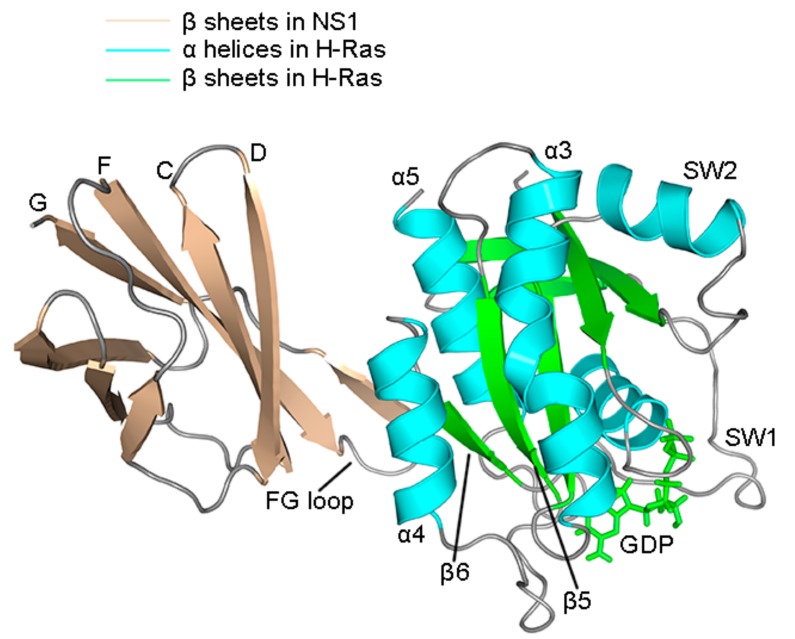
Cartoon structure of H-Ras–NS1 complex, with the major secondary structures specified. SW1 and SW2 represent the Switch 1 and 2 regions, respectively. β sheets in NS1 and H-Ras are colored in wheat and green, respectively. α helices in H-Ras are colored in cyan and loops are in gray.

There was more anticorrelation in the H-Ras^R135K^–NS1 complex. In wild type (Figure 2A), A1 represented the anticorrelation between the G β sheets and FG loop in NS1 and the α4 helix and β6 sheet in H-Ras (Figure 3), while A2 showed that the G β sheet and FG loop also anticorrelated with the β5 sheet and α3 helix in H-Ras. The anticorrelation between F β sheet and β5 sheet was portrayed by A3, and A4 together with A5 depicted the anticorrelation between two groups of residues within NS1, implying a potential allosteric network. In the mutated system, all the anticorrelation relationships described above were preserved, and remarkably, they all strengthened. Additionally, new anticorrelation was found between the C and D β sheet in NS1 and β5 sheet as well as α3 helix in H-Ras (A6 in Figure 2B). Moreover, more residues became anticorrelated with each other within the chain of NS1 or H-Ras. The newly formed anticorrelation indicated that the mutation disrupted the residues interaction network throughout the complex (A7–A11 in Figure 2B). 

The correlation motions within the wild type and R135K H-Ras–NS1 complexes were similar, but they were enhanced after mutation. In wild type, C1 to C3 all showed the strong correlation in local regions, while C4 portrayed the correlation of the residues within the α4 helix structure. Several residues lying along the binding surface of H-Ras correlated with residues in the bottom of F and G β sheets and the FG loop in NS1, as was shown by C5. After mutation, intra-molecular correlation in NS1 did not change significantly, but the ones in H-Ras were much stronger. Besides C4, local correlation was established within the β5 sheet and α3 helix respectively (C6 and C7 in Figure 2B correspondingly). Two new correlations, C8 and C9, represented the correlation of α4-α3 and SW2-α3, respectively. Moreover, in addition to the strengthened C5, correlation along the interface became more significant after the mutation in spite of their relatively weak magnitudes. Collectively, the increase in correlation reflected the fact that there was more interaction in the mutation system, which suggested that the whole system became more elastic. More fluctuation emerged as a result of the increased flexibility, especially for the interface part.

**Figure 4:**
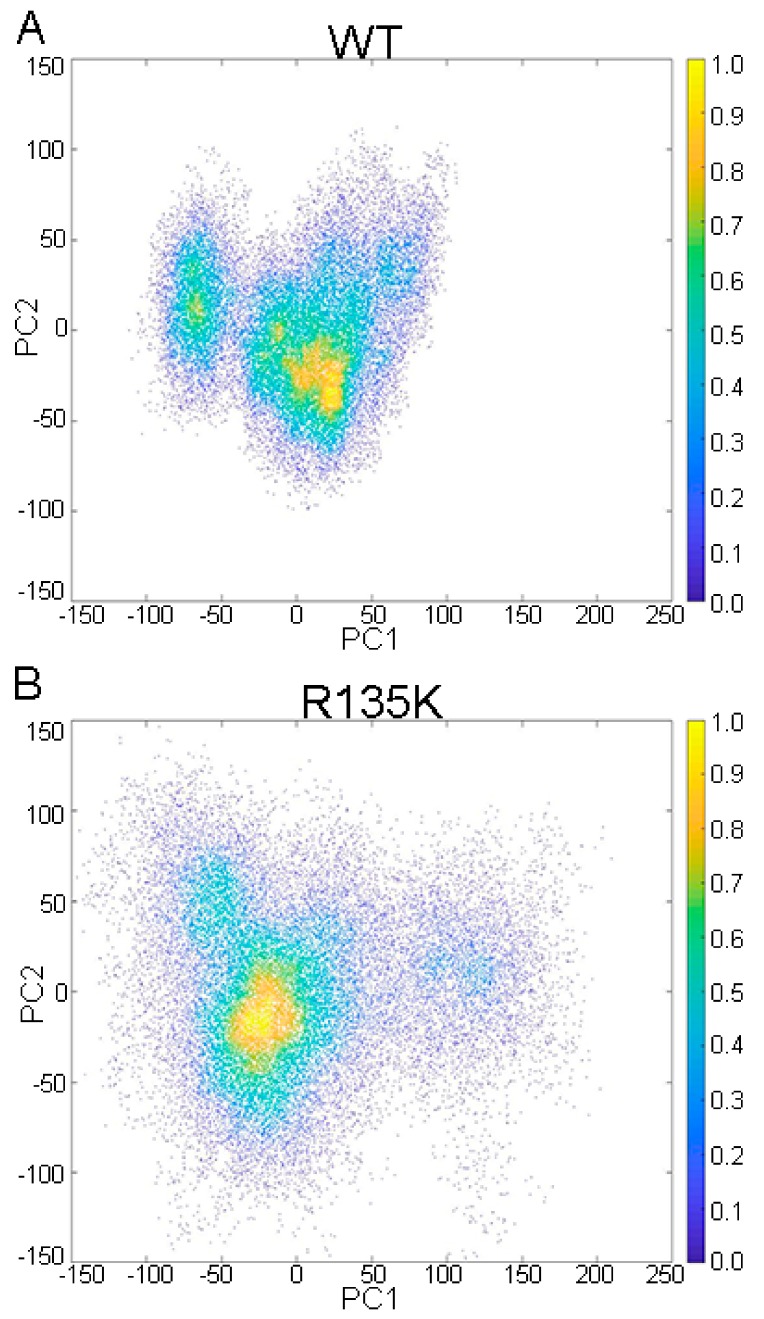
Projections of trajectories of the wild type (**A**) and R135K (**B**) H-Ras–NS1–GDP complexes onto the corresponding first two principal components (PC1 and PC2).

Besides DCCM, principal component analysis (PCA) was also carried out to characterize the major motions and fluctuations of the wild type and R135K system. As shown in Figure 4, the major conformation of these two systems was similar to each other, which was consistent with the results from RMSD calculation. However, the distribution of the conformation of the mutated system was broader than the wild type. This implied that there was more freedom and fluctuation in the R135K system, consistent with the RMSF results. Therefore, both DCCM and PCA analysis suggested a more flexible and less constrained R135K H-Ras–NS1 system.

### 2.3. MM/GBSA Free Energy Analysis

To clarify the energetics of the binding of NS1 quantitatively, we carried out the MM/GBSA calculations. The binding free energy (*ΔG_binding_*) of NS1 to the H-Ras–GDP complex was obtained as results for the wild type and mutated system, respectively (Table 1). As was shown by the MM/GBSA analysis, the *ΔG_binding_* for wild type H-Ras was −55.19 ± 7.88 kcal/mol, while for the mutated system, the result was −38.47 ± 8.56 kcal/mol. A higher binding free energy for the mutated system showed that the NS1 binding was not as favored as it was in the wild type, and the result was consistent with the in vitro experiments [40]. A detailed analysis of the energy contributions showed that it was electrostatic force that was mainly responsible for the increase in the free energy for NS1 binding, because the difference between this parameter before and after mutation increased most significantly, while others almost remained unchanged or decreased instead. Moreover, the unfavorable contribution to binding in the mutation system also stemmed from the increase in the gas free energy (*ΔG_gas_*), which exhibited a significant rise.

In order to reveal the underlying molecular mechanisms of the decrease in binding free energy after mutation, we employed PISA (Proteins, Interfaces, Structures and Assemblies) [55] to analyze the interactions within the wild type and R135K mutated systems. Hydrogen bonds and salt bridges within two systems were uncovered as results. In the wild type complex, there existed 17 hydrogen bonds and 9 salt bridges in the interface, while after mutation, only 13 hydrogen bonds and 6 salt bridges remained. Specifically, we focused on these interactions involving the mutated site, and we found that R to K mutation also led to the reduced molecular interactions on it (Table 2).

**Figure 5:**
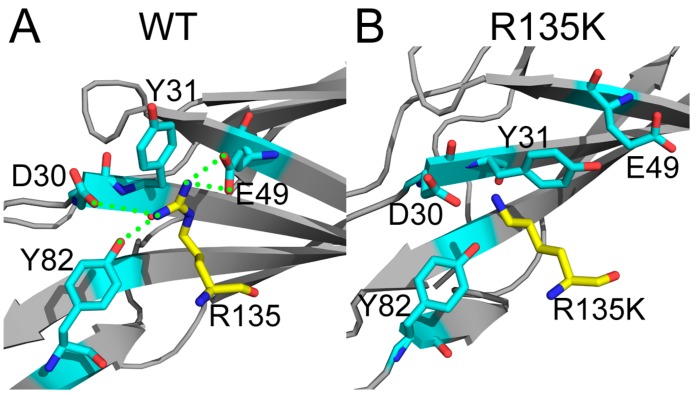
Detailed interaction around mutation site in wild type system (**A**); and R135K system (**B**). R135(K) in H-Ras was in yellow, and important residues in NS1 interacting with R135 were in cyan. Inter-molecular hydrogen bonds were depicted by green dashed lines, and the overall structures of NS1 were in gray. For clarity, non-polar hydrogen atoms were omitted.

Between NS1 and H-Ras, R135 formed 4 hydrogen bonds and 5 salt bridges in the wild type complex, both of which were highest among all residues, implying its critical role in NS1’s binding to H-Ras (Figure 5A). Nevertheless, in the mutated system, all the hydrogen bonds were lost, and only 2 salt bridges were preserved, suggesting a weak interaction in the mutant (Figure 5B). Moreover, besides hydrogen bonds and salt bridges, in the wild type complex, a cation-pi interaction was formed between R135 of H-Ras and Y31 of NS1. However, after mutation such interaction was lost due to conformation changes of these two residues, especially the movement of the phenyl group in Y31 of NS1 (Figure 5). In H-Ras, R135K mutation affected the conformation of the mutation site as well as the surrounding residues, which led to reduced inter-molecular interactions. Loss in hydrogen bonds, salt bridges, and cation-pi were convincing reasons for the NS1 unbinding, and it could also explain the sharp increase in the binding reaction free energy.

**Figure 6:**
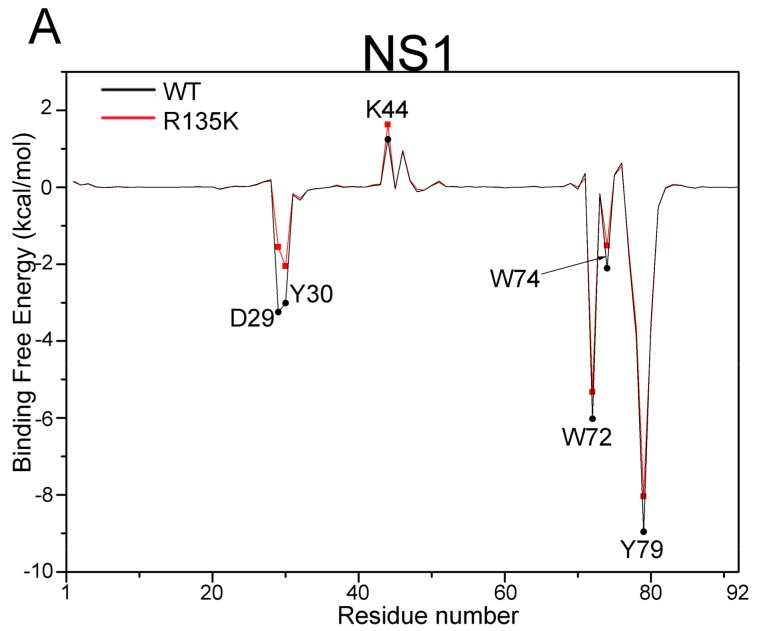

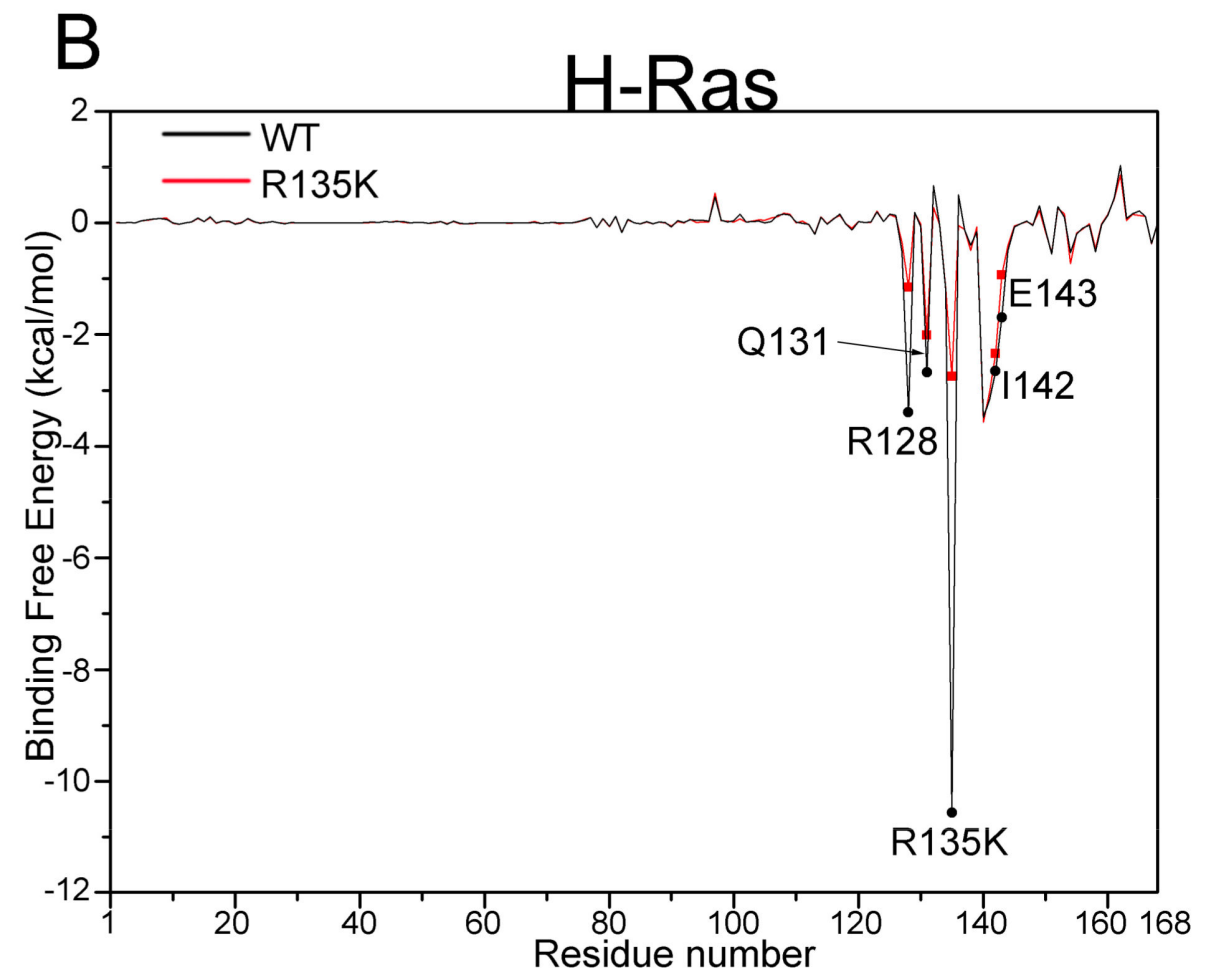
Free energy contributions to the binding of NS1 to H-Ras decomposed into each residue corresponding to the NS1 (**A**) and H-Ras (**B**). The residues whose energy contribution was greater after mutation for more than 0.3 kcal/mol were specified respectively.

**Figure 7:**
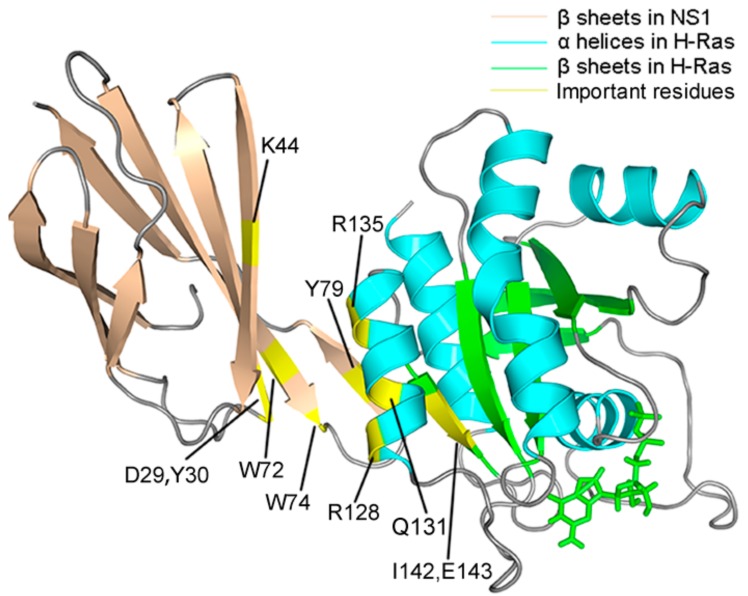
A snapshot of the structure of the interface between NS1 and H-Ras, with the important residues mentioned above highlighted (colored in yellow).

To quantify the contributions of each residue to free energy change in detail, we decomposed the total binding free energy into each residue (Figure 6), and the ones that contributed to the increase of the binding free energy in the mutation system for at least 0.3 kcal/mol were specified (Table 2). Residues whose contribution to binding free energy increased more than 0.3 kcal/mol included D29, Y30, K44, W72, W74 and Y79 from NS1, and R128, Q131, R135K, I142 and E143 from H-Ras. Most of these residues were distributed along the interface between NS1 and H-Ras (Figure 7). W74 and Y79 in NS1 both situated on the interface between NS1 and H-Ras, while W72, K44, Y30 and D29 all located farther from the interface, implying a possible allosteric effect involved in the NS1 binding. As for H-Ras, all of the residues specified located along the α4-β6-α5 interface, with R135K, Q131, and R120 in α4 helix and I142 and E143 in β6 sheet. Although for most of the residues in the complex structure, no significant free energy difference was observed, there were still some regions transformed into more flexible and less stable states. A more than 0.3 kcal/mol increase in free energy contribution reflected that the residues mentioned above existed in a higher energy state, which was unstable and negatively affected the binding of NS1 to H-Ras, and it was these local changes in energy states that were responsible for the disassociation of NS1 from H-Ras. Interestingly, the R135K mutation did not influence the nearby residues, and instead, its negative effect was coupled with several “remote” residues, at least 4 residues away. Such long-range effect and the coupling implied that there was topological linkage among these residues, and the mechanism underlying the effect of the mutation was allosteric.

### 2.4. Superposition of the Wild Type and Mutation Structures

To visualize the conformation changes between the wild type and R135K mutation complex, structures of these two complexes after 200-ns MD simulation were superimposed. For H-Ras, most of the protein was only slightly changed, and significant conformational alterations were mainly observed on the binding surface of NS1, especially for the helices. As for the NS1, there were more structural changes due to its monobody nature, but the most obvious ones were also found along the interface. 

**Figure 8:**
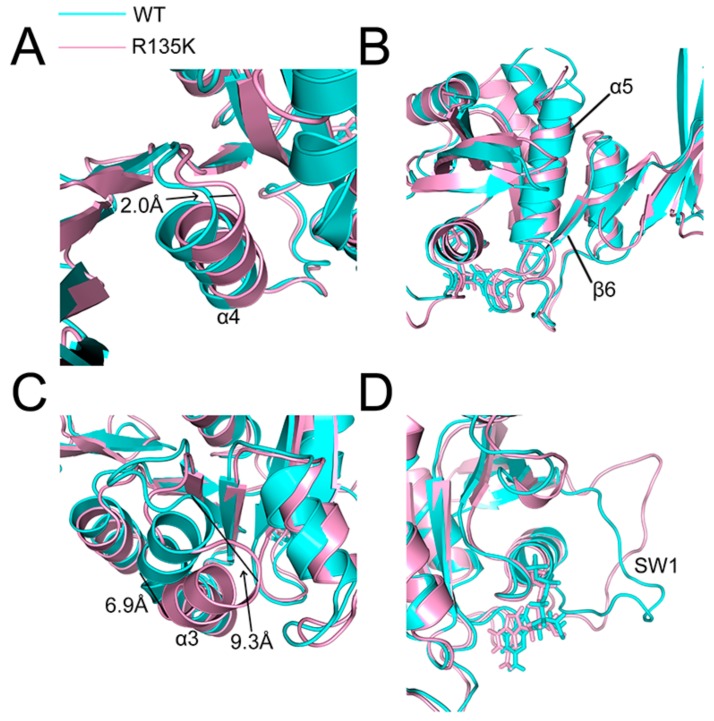
Detailed superposition structures analysis of α4 helix (**A**); α5 helix and β6 sheet (**B**); α3 helix (**C**) and SW1 (**D**). Wild type H-Ras–NS1 complex is colored in cyan, while R135K H-Ras–NS1 complex is in pink.

In the α4-β6-α5 sandwich structure, α4 helix of H-Ras in the R135K mutant moved backward for about 2.0 Å compared to that in the wild type (Figure 8A). The α5 helix slightly shifted away from the interface, while β6 sheet was not significantly influenced by the mutation (Figure 8B). Similar with the α4 helix, the α3 helix altered significantly, particularly for its top, shifting for at least 6.0 Å (Figure 8C). Near the α3 helix, SW2 in the mutated state moved slightly away, but the whole structure was generally unchanged. In contrast, as a loop, SW1 was significantly affected by the mutation. It changed irregularly, and took on a completely different conformation (Figure 8D). Given the significant conformational changes induced by the R135K mutation, it was reasonable that there was an allosteric network within the H-Ras structure, and the point mutation disrupted this network, especially for the part near the binding interface, and therefore negatively affected the interaction between NS1 and H-Ras.

### 2.5. Allosteric Network Analysis

To demonstrate that R135K mutation disrupted the original allosteric network within H-Ras, and to identify the signal propagation pathways, dynamics network analysis was carried out with the NetworkView plugin in VMD [56]. This process produced the shortest (optimal) signaling pathways and the suboptimal pathways as results. Analysis focused on the mutation site and the residues whose binding free energy were significantly affected by R135K mutation (an increase greater 0.3 kcal/mol). The results are shown in Table 3.

The optimal pathways between two selected residues would be considered as the potential allosteric pathway, and for both the optimal and suboptimal pathways, the residues involved were revealed. In such analysis, shorter length of the optimal pathway, less involved residues, and larger number of suboptimal pathways all indicated a stronger allosteric relationship between the two chosen residues. In Table 4, the first two pathways, R135-R128, and R135-Q131 all displayed these characteristics, and hence for these two pairs of residues, the allosteric signal between them was more intense in the wild type complex. On the other hand, despite the phenomena that the length of the R135(K)-I142 and R135(K)-E143 paths were slightly longer in the wild type system compared with the mutant, combined with the fact that in the wild type complex, less residues were involved, and more suboptimal pathways existed, it still suggested that the allosteric signaling was stronger in the wild type system. Therefore, it could be concluded that the allosteric pathways connecting these residues were disrupted by the R135K mutation. 

The products of NetworkView, the optimal pathways in the wild type system are shown as follows:

R135→Q131→R128

R135→Q131

R135→A134→Y141→I142

R135→A134→Y141→E143

None of these pathways were preserved in the mutation system, which suggested that the point mutation at the starting point of these pathways significantly influenced the original allosteric relationship within the wild type complex, and since the destination of these pathways all located on the interface of the binding of NS1 towards H-Ras, it was not surprising that the binding affinity was severely negatively affected by the mutation. Moreover, the result above implied that R135 might be a critical node within the topological network of H-Ras, which would be worth further study. Hence, the unfavored binding of NS1 in R135K system was a result of the disruption of the allosteric pathways due to the mutation at the critical node of the allosteric network.

## 3. Materials and Methods

### 3.1. Construction of Simulation System

The crystal structure of the H-Ras and NS1 and GDP complex at 1.4 Å (PDB ID: 5E95) [40] was obtained from the RCSB Protein Data Bank [57]. Point mutation at number 135 amino acid was introduced using Maestro 10.2 (Maestro, Schrödinger, LLC, New York, NY, USA), and the original arginine was mutated into lysine.

### 3.2. MD Simulations

Amber 14 [58] was employed for MD simulations for the wild type complex and the R135K mutated complex. The force field parameters of the complex system were calculated based on the Amber ff03 force field and the general amber force field (GAFF). The H-Ras–NS1–GDP complex was first solvated using the TIP3PBOX water model, and then sodium ions were added to neutralize the whole system. After these preparations, the complex system underwent two rounds of energy minimization. In the first step, the macro-molecules scaffold was held rigid, and the energy of the water molecules and counterions was minimized after 5000 steps of maximum minimization cycles, and in the second round, the whole system was relaxed and underwent minimization without any restriction. After minimizing energy, the whole system was heated from 0 to 300 K within 300 ps, under a positional restraint of 10 kcal/(mol Å^2^) in a canonical ensemble (NVT). Then equilibration of the system was carried out at 300 K, also with a 10 kcal/(mol Å^2^) positional restraint, in a canonical ensemble (NVT) for 700 ps. Finally, 200 ns MD simulation was performed for both the wild type and mutated system in isothermal and isobaric ensemble with periodic boundary conditions. The particle mesh Ewald method [58] was employed to analyze the long-range electrostatic interactions and a cutoff of 10 Å was applied to treat the short-range electrostatics and van der Walls interactions. All covalent bonds involving hydrogen atoms were restricted by the SHAKE method, and the final trajectories were written out every 5 ps.

### 3.3. Principal Component Analysis

Principal component analysis (PCA) was carried out with the help of the *cpptraj* plugin of the Amber [59]. Equation (1) was used to calculate the covariance matrix *Z* of the complex system:

(1) $$Z_{ij}=\langle \left(x_{i}-\langle x_{i}\rangle \right)\left(x_{j}-\langle x_{j}\rangle \right)\rangle \left(i,j=1,2,3,\ldots 3N\right)$$

where *x_i_* stands for the Cartesian coordinate of the Cα atom at the number *i*, $\langle x_{i}\rangle$ represents the time average over the selected configurations in the trajectories, and *N* represents the total number of Cα atoms.

### 3.4. Molecular Mechanics Generalized Born Surface Area Calculations

Molecular Mechanics Generalized Born Surface Area (MM/GBSA) calculation was carried out using the MMPBSA.py plugin. Free energy for the complex system, receptor (GDP-bound H-Ras), and ligand (NS1) was calculated respectively, and the total free energy difference for the ligand binding was given by the following Equation (2):

*ΔG = G_complex_ − G_receptor_ − G_ligand_* (2)

In Equation (2), the free energy terms equaled to the sum of the gas phase molecular mechanical energy (*ΔE_gas_*) and the solvation free energy (*ΔG_solvation_*) and the entropy term (*−TΔS*):

*ΔG = ΔE_gas_ + ΔG_solvation_ − TΔS* (3)

*ΔE_gas_* originated from the van der Waals energy (*ΔE_vdW_*), electrostatic energy (*ΔE_ele_*) and gas phase internal energy (*ΔE_int_*):

*ΔE_gas_ = ΔE_vdW_ + ΔE_ele_ + ΔE_int_* (4)

The solvation free energy, obtained by the continuum solvent methods, were divided into the polar contribution (*ΔG_PB/_*) and the non-polar contribution (*ΔG_nonpolar_*):

*ΔG_solvation_ = ΔG_PB/_ + ΔG_nonpolar_* (5)

The finite difference PB model was used to calculate the electrostatic solvation energy. 1 and 80 were chosen as the interior (solute) and exterior (water) dielectric constants respectively. The non-polar contribution (*ΔG_nonpolar_*) to the solvation free energy (*ΔG_solvation_*) was calculated according to the solvent-accessible surface-area with Equation (6):

*ΔG_solvation_ = γSASA + b* (6)

SASA represented the solvent-accessible surface-area, solvation parameter γ was 0.00542 kcal (mol^−1^ Å^−2^) and another solvation parameter b was 0.92 kcal/mol. The conformation entropy (*−TΔS*) was usually calculated by normal mode analysis with quasi harmonic model, but it could be omitted here due to the relative low RMSD of two complex systems. Additionally, NS1 is a protein and computation of the conformation entropy for the protein-protein interactions represents a challenging. Considering the potential difficulties and the similar overall structural mode, we only focused on the relative ordering of the free energy changes without calculation of the *−TΔS* term. Decomposition of the binding free energy into residues interaction pairs was carried out using the MM/GBSA method. The binding energy of each interaction pair consisted of three parts: *ΔE_vdW_*, *ΔE_ele_*, and *ΔG_GBSA_.*

### 3.5. Dynamic Network Analysis

Dynamic cross-correlation matrix (DCCM), which revealed the correlation between two residues, was produced from the *cpptraj* plugin. The Cα were selected as the representative of every residue, and the cross-correlation coefficient *X_i_*_,*j*_ for Cα pairs were calculated by Equation (7):

(7) $$X_{ij}=\frac{\langle \left(r_{i}-\langle r_{i}\rangle \right)\cdot \left(r_{j}-\langle r_{j}\rangle \right)\rangle }{\sqrt{{\langle \left(r_{i}-\langle r_{i}\rangle \right)\rangle }^{2}{\langle \left(r_{j}-\langle r_{j}\rangle \right)\rangle }^{2}}}$$

The products of Equation (7) were then applied to analysis the residues network of the complex topologically. One of the plugin of VMD, NetworkView [56], was used for the topological network analysis of the complex to explore the allosteric network in the protein and monobody system. Every residue in the complex, represented by their Cα, was regarded as a node in the topological network, and edges were drawn between these nodes if they were related to each other. The cross-correlation coefficient *X_i_*_,*j*_ was used to calculate the distance (*d_ij_*) of the edges between nodes, with the length equaling the product of Equation (8):

*d_ij_* = −log(|*X_i_*_,*j*_|) (8)

in which *i*, *j* stood for two nodes, and *X_i_*_,*j*_ was given by Equation (7). Additionally, suboptimal pathway was also calculated. All pathways within in a distance of 20 to the shortest (optimal) pathway were analyzed, and the number of the suboptimal pathways and the residues involved in these pathways most frequently were produced as results.

## 4. Conclusions

In the present study, we explored the unbinding of NS1 to H-Ras caused by R135K mutation using MD simulations and dynamic network analysis. The overall conformation of the complex was not significantly influenced by the mutation, but some regional changes occurred, especially in the monobody-protein interface. Most of hydrogen bonds, salt bridges and cation-pi interaction at the interface in the wild type complex were disrupted by the R135K mutation, and the critical residues responsible for binding were identified. Furthermore, the identified allosteric pathways in the wild type were also disrupted by the R135K mutation. These collective results resulted in the unbinding of NS1 to H-Ras induced by the mutation. Our discovery of the critical role of R135 in H-Ras allosteric network and the detailed monobody binding mechanisms provided structural basis for the optimization of NS1, and offered a guidance for future development of drugs targeting Ras. Protein-protein interactions are important targets in drug discovery [60,61,62], and recently, peptidomimetics have become a new direction in targeting protein-protein interactions [63]. With the hotspot residues for NS1-H-Ras interaction identified in our study, monobody NS1 could be optimized and related peptidomimetics modulators could be designed based on the discovery here. Moreover, the findings of the allosteric network also shed light on regulating the Ras protein through allostery. 

## Figures and Tables

**Table 1:** Free energy analysis (kcal/mol) for the binding of NS1 to H-Ras–GDP complex ^a^.

Items	WT	R135K	Energy Difference
*ΔE_vdW_* ^b^	−68.36 (4.36)	−61.57 (5.49)	6.79
*ΔE_ele_* ^c^	−75.44 (31.88)	−37.60 (39.73)	37.84
*ΔE_GB_* ^d^	97.24 (28.18)	68.16 (35.80)	−29.08
*ΔE_SURF_* ^e^	−8.63 (0.57)	−7.46 (0.78)	1.17
*ΔG_gas_*	−143.80 (32.67)	−99.17 (40.27)	44.63
*ΔG_nonpolar_*	97.37 (1.22)	68.74 (10.14)	−28.63
*ΔG_polar_*	−8.76 (0.13)	−8.04 (0.12)	0.72
*ΔG_binding_*	−55.19 (7.88)	−38.47 (8.56)	16.72

^a^ Numbers in the parentheses are the standard deviations; ^b^ Energy contribution from the van der Waals force; ^c^ Energy contribution from the electrostatic force; ^d^ Energy contribution from the Generalized-Born term; ^e^ Energy contribution from the solvent-accessible surface term.

**Table 2:** Summary of the hydrogen bonds and salt bridges between R135 and NS1 in wild type (up) and R135K (down) H-Ras–NS1.

**Number**	**H Bonds or Salt Bridges**		**Distance (Å)**
Hb1	NS1-D30-OD2	H-Ras-R135-NH2	3.75
Hb2	NS1-E49-OE1	H-Ras-R135-NE	2.73
Hb3	NS1-E49-OE2	H-Ras-R135-NH1	2.89
Hb4	NS1-Y82-OH	H-Ras-R135-NH2	2.91
Sb1	NS1-D30-OD2	H-Ras-R135-NH2	3.75
Sb2	NS1-E49-OE1	H-Ras-R135-NE	2.73
Sb3	NS1-E49-OE1	H-Ras-R135-NH1	3.49
Sb4	NS1-E49-OE2	H-Ras-R135-NE	3.67
Sb5	NS1-E49-OE2	H-Ras-R135-NH1	2.89
**Number**	**Salt Bridges**		**Distance (Å)**
Sb1	NS1-E49-OE1	H-Ras-K135-NZ	3.76
Sb2	NS1-E49-OE2	H-Ras-K135-NZ	3.96

**Table 3:** Free energy contribution (kcal/mol) by residue and the corresponding free energy difference of H-Ras–NS1–GDP ^a^.

	Residue	WT	R135K	Energy Difference ^b^
NS1	D29	−3.25 (2.59)	−1.56 (2.39)	1.69
	Y30	−3.02 (0.48)	−2.05 (0.77)	0.97
	K44	1.24 (0.65)	1.62 (1.39)	0.38
	W72	−6.02 (0.68)	−5.32 (0.73)	0.70
	W74	−2.11 (0.91)	−1.52 (0.88)	0.59
	Y79	−8.96 (0.87)	−8.04 (1.07)	0.92
H-Ras–GDP	R128	−3.40 (2.33)	−1.15 (2.38)	2.25
	Q131	−2.68 (1.36)	−2.01 (1.21)	0.67
	R135K	−10.56 (1.94)	−2.75 (3.27)	7.81
	I142	−2.65 (0.76)	−2.34 (0.76)	0.31
	E143	−1.70 (1.09)	−0.94 (1.29)	0.76

^a^ Number in the parentheses are the standard deviations; ^b^ Energy difference was calculated by *G_R135K_-G_WT_.*

**Table 4:** Allosteric pathways analysis between R135K and residues important for NS1 binding.

Pathway	Length ^a^		Residue ^b^		Subopt ^c^	
	WT	R135K	WT	R135K	WT	R135K
R135(K)-R128	38	60	3	8	12	1
R135(K)-Q131	21	29	2	5	1	1
R135(K)-I142	68	61	4	8	9	0
R135(K)-E143	86	84	4	9	12	1

^a^ The length of the shortest pathway of the corresponding residues; ^b^ The number of residues involved in the shortest pathway; ^c^ The number of suboptimal pathways.
